# Loss of the insulin receptor in murine megakaryocytes/platelets causes thrombocytosis and alterations in IGF signalling

**DOI:** 10.1093/cvr/cvv132

**Published:** 2015-04-22

**Authors:** Samantha F. Moore, Christopher M. Williams, Edward Brown, Thomas A. Blair, Matthew T. Harper, Richard J. Coward, Alastair W. Poole, Ingeborg Hers

**Affiliations:** 1 School of Physiology and Pharmacology, School of Medical Sciences, University of Bristol, University Walk, Medical Sciences Building, BristolBS8 1TD, UK; 2 School of Clinical Sciences, Dorothy Hodgkin Building, University of Bristol, BristolBS1 3NY, UK

**Keywords:** Platelets, Insulin, Glycoprotein VI, IGF, Insulin resistance

## Abstract

**Aims:**

Patients with conditions that are associated with insulin resistance such as obesity, type 2 diabetes mellitus, and polycystic ovary syndrome have an increased risk of thrombosis and a concurrent hyperactive platelet phenotype. Our aim was to determine whether insulin resistance of megakaryocytes/platelets promotes platelet hyperactivation.

**Methods and results:**

We generated a conditional mouse model where the insulin receptor (IR) was specifically knocked out in megakaryocytes/platelets and performed *ex vivo* platelet activation studies in wild-type (WT) and IR-deficient platelets by measuring aggregation, integrin α_IIb_β_3_ activation, and dense and α-granule secretion. Deletion of IR resulted in an increase in platelet count and volume, and blocked the action of insulin on platelet signalling and function. Platelet aggregation, granule secretion, and integrin α_IIb_β_3_ activation in response to the glycoprotein VI (GPVI) agonist collagen-related peptide (CRP) were significantly reduced in platelets lacking IR. This was accompanied by a reduction in the phosphorylation of effectors downstream of GPVI. Interestingly, loss of IR also resulted in a reduction in insulin-like growth factor-1 (IGF-1)- and insulin-like growth factor-2 (IGF-2)-mediated phosphorylation of IRS-1, Akt, and GSK3β and priming of CRP-mediated platelet activation. Pharmacological inhibition of IR and the IGF-1 receptor in WT platelets recapitulated the platelet phenotype of IR-deficient platelets.

**Conclusions:**

Deletion of IR (i) increases platelet count and volume, (ii) does not cause platelet hyperactivity, and (iii) reduces GPVI-mediated platelet function and platelet priming by IGF-1 and IGF-2.

## 1. Introduction

Platelets are small anucleate cells that are formed from megakaryocytes, and their primary function is to regulate haemostasis.^1^ Platelets normally circulate in a resting discoid state, maintained by factors released from an intact endothelium. When a vessel is damaged an underlying matrix of proteins including collagen are exposed, to which the platelets are able to rapidly adhere. This will lead to platelet activation that includes (i) secretion of mediators from dense and α-granules that can activate and recruit platelets to the site of injury, (ii) activation of the integrin α_IIb_β_3_ which causes platelets to aggregate through bridging interactions with fibrinogen, and (iii) promotion of coagulation reactions that produce thrombin to further amplify activation. The resulting primary platelet plug will limit blood loss. Dysregulation of these processes leads to platelet hyperactivity promoting aberrant thrombosis. Platelet hyperactivity and a concurrent increased risk in thrombotic complications are often reported in patients with obesity, type 2 diabetes mellitus (T2DM), polycystic ovary syndrome, high blood pressure, and abnormal cholesterol levels.^2–4^ One of the common factors in these diseases is insulin resistance, a condition where cells have a reduced ability to respond to insulin.

Insulin, insulin-like growth factor-1 (IGF-1), and insulin-like growth factor-2 (IGF-2) are related peptide hormones that mediate their actions through the structurally similar receptors, including insulin receptor (IR) and IGF-1-receptor (IGF1R).^5–7^ These receptors are composed of two monomers comprising an extracellular α-subunit and a transmembrane β-subunit.^8^ Ligand binding induces receptor autophosphorylation at various tyrosine residues, recruitment of IR substrates (IRS), and activation of mitogen-activated protein kinase (MAPK) and phosphoinositide 3-kinase (PI3K).^9^ In the case of insulin, which only has high affinity for IR,^10^ activation of these signalling pathways promote downstream processes involved in regulating blood glucose levels.^11^ A loss of insulin signalling initially results in increased insulin production, but eventually leads to hyperglycaemia and development of T2DM when the body can no longer compensate for the lack of effect of insulin.

Platelets from insulin-resistant patients show signs of hyperactivity including (i) reduced responsiveness to the factors released by the endothelium,^12^ (ii) increased responsiveness to platelet agonists,^13^ (iii) increased levels of the α-granule marker P-selectin (CD62P),^14^ and (iv) increased expression of integrin α_IIb_β_3_.^15^ One proposed mechanism for the observed increase in platelet reactivity in insulin-resistant patients is a reduction in IR signalling. The latter could be through insulin resistance of megakaryocytes and/or platelets themselves.^16–18^ A widely accepted approach to study the effect of insulin resistance in different tissues is the conditional removal of the IR itself,^19^ which results in the complete ablation of insulin signalling. This approach has been instrumental in investigating the pathogenesis of insulin resistance and diabetes.^20–22^ Here, we determined whether platelet hyperactivity in insulin-resistant patients can be attributed to a loss of IR signalling in megakaryocytes and/or platelets, by investigating platelet function in platelet/megakaryocyte-specific IR knockout mice (IR KO).

## 2. Methods

### 2.1 Mice

Animal studies were approved by local research ethics and mice bred for this purpose under a UK Home Office project license (PPL30/2908). To investigate the role of IR expression and function in platelets, mice homozygous for a floxed IR allele in which *loxP* sites flank exon 4 of IR gene^22^ were crossed with transgenic mice in which expression of *Cre* recombinase was driven by the *Pf4* gene promoter.^23^ IR^flox/flox^ *Pf4*-Cre mice (C57BL/6) were obtained in a Mendelian ratio and were healthy with no growth abnormalities. IR^flox/flox^ *Pf4*-Cre^+^ mice are referred to as KO and age-/sex-matched IR^flox/flox^ *Pf4*-Cre^−^ littermate controls as wild type (WT).

### 2.2 Reagents

IRS-1, IRS-2, p85, Akt, pSer^473^ Akt, pGSK3β^S9^ PKC phospho-motif, pTyr^191^ linker of activated T cells (LAT), pTyr^416^ Src, pTyr^525/526^ Syk, pTyr^759^ PLCγ2, LAT, Syk, PLCγ2, and SLP-76 antibodies were from Cell Signalling Technologies (New England Biolabs, Hitchin, UK). IR (C-19), IGF1R (C-20), IRS-3 (R-190), and GAPDH antibodies were from Santa Cruz (Insight Biotechnology, Wembley, UK). The pTyr^128^ SLP-76 antibody and Retic-count™ were from BD Biosciences (Oxford, UK). The anti-phosphotyrosine antibody 4G10 was from Millipore (Watford, UK). FITC-conjugated glycoprotein VI (GPVI, JAQ1) anti-mouse antibody was from Emfret Analytics (Wurzberg, Germany). PE-conjugated CD61 (HM beta 3.1) was from AbD Serotec (Kidlington, UK). Chronolume was from Chrono-log Corporation (Labmedics, Manchester, UK). IGF-1 and IGF-2 were from Immunological and Biochemical Test Systems (Binzwangen, Germany). Cross-linked collagen-related peptide (CRP-XL) was synthesized by Prof. Richard Farndale (Department of Biochemistry, University of Cambridge, UK). The PAR-4 agonist (PAR4-activating peptide, PAR4-AP, and AYPGKF-NH_2_) was synthesized by Peptide Synthetics Peptide Protein Research Ltd (Hampshire, UK). NVP-AEW541 was from Caymen Chemical (Cambridge Bioscience, Cambridge, UK). IGF-1 mouse/rat quantikine ELISA kit was from R&D Systems™ (Abingdon, UK). IGF-2 mouse ELISA kit was from Abcam^®^ (Cambridge, UK). Microcystin-LR was from Axxora (Nottingham, UK). Enhanced chemiluminescent detection reagents were from GE Healthcare (Bucks, UK). Peroxidase-conjugated secondary antibodies were from Jackson Immunoresearch (Stratech, Newmarket, UK). NuPAGE SDS–PAGE sample buffer was from Invitrogen (Paisley, UK). All other reagents were from Sigma (Poole, UK), unless otherwise indicated.

### 2.3 Platelet isolation

Mice (8–16 weeks old) were sacrificed by rising CO_2_ inhalation, in accordance with Schedule 1 of the Animals (Scientific Procedures) Act (1986), and blood was drawn by cardiac puncture into a syringe containing 4% trisodium citrate (1 : 10, v/v). Femurs were removed for megakaryocyte quantification experiments. Prior to platelet preparation, complete blood counts were conducted (Pentra ES60, Horiba) and adjusted for anticoagulant volume. Washed platelets were isolated as previously described.^24^ Platelets were resuspended at 4 × 10^8^/mL in modified HEPES-Tyrode's buffer [145 mmol/L NaCl, 3 mmol/L KCl, 0.5 mmol/L Na_2_HPO_4_, 1 mmol/L MgSO_4_, 10 mmol/L HEPES, pH 7.2, 0.1% (w/v) d-glucose, 0.02 U/mL apyrase, and 10 μmol/L indomethacin].

### 2.4 Megakaryocyte quantification

Murine femurs were immersion-fixed for 1 h in 2.5% glutaraldehyde in 0.1 M sodium cacodylate buffer. Both ends of the femur were cut and the marrow flushed out as a whole piece of tissue by gently flowing through fixative using a 21-gauge needle. This was immersed in fresh fixative for a further hour. The marrow was post-fixed with 1% osmium tetroxide and stained with 1% uranyl acetate before dehydration through a graded series of ethanol. The tissue was washed three times with propylene oxide and left rotating overnight in a 1 : 1 mixture of propylene oxide and Epon. Propylene oxide was allowed to evaporate over 4 h before the marrow was transferred into fresh Epon and rotated overnight. The tissue was then mounted in moulds and left to harden at 60°C for 48 h. Thick sections (500 nm) of the entire length of bone marrow were taken at 20 µm intervals. Sections were stained with toluidine blue and mounted on coverslips. A stitched tile scan of the entire bone marrow area was acquired using an Leica DMI600 inverted microscope. Megakaryocytes were positively identified based on size, morphology, or ploidy and quantified using ImageJ. For TEM imaging, ultrathin sections (70 nm) were taken and stained with uranyl acetate followed by lead citrate. Images were acquired on a Tecnai 12 (FEI).

### 2.5 Aggregation and ATP secretion

Platelet aggregation and dense granule secretion were monitored as previously described.^25^ Briefly, washed platelets (2 × 10^8^/mL) were stimulated with agonist while simultaneously monitoring aggregation and dense granule secretion using a luciferin/luciferase reagent (Chronolume) in a Chronolog 590-2A aggregometer at 37°C under stirring conditions.

### 2.6 Flow cytometry

Two colour analysis of platelet activation was performed as previously described using a PE-conjugated antibody directed against the high affinity form of integrin α_IIb_β_3_ (JON/A) and a FITC-conjugated antibody for the α-granule marker CD62P (Wug.E9).^25^ Platelets (2 × 10^7^/mL) were stimulated with PAR4-AP or CRP-XL in the presence of PE-JON/A and FITC-CD62P for 10 min. GPVI surface expression was assessed by incubating resting platelets with a FITC-conjugated GPVI (JAQ1), anti-mouse antibody for 10 min. All samples were fixed in 1% paraformaldehyde and analysed on BD LSR II (BD Bioscience) using the FACSDiva software (10 000 platelet events per sample). For reticulated platelet quantification, paraformaldehyde-fixed blood was diluted 1 : 50 and stained with PE-CD61 antibody before incubation with thiazole orange solution (Retic-count™, BD Biosciences, Oxford, UK) for 1 h. Samples were read on BD LSR II (BD Bioscience) with platelets identified by their logarithmic side scatter (SSC) and CD61 positivity expression. The amounts of reticulated platelets are expressed as the percentage of thiazole orange and CD61-PE double-positive cells in 10 000 identified platelets.

### 2.7 Protein extraction, immunoprecipitation and immunoblotting

Platelets (4 × 10^8^/mL) were incubated with insulin, IGF-1, CRP-XL, or PAR4-AP as indicated. Platelet suspensions were either lysed directly in 4× NuPAGE sample buffer (whole cell lysate) or extracted with an equal volume of ice-cold lysis buffer [50 mmol/L Tris pH 7.5, 240 mmol/L NaCl, 2 mmol/L EDTA, 2% (v/v) IGEPAL CA-630, 40 mmol/L sodium β-glycerol phosphate, 2 mmol/L benzamidine, 2 μmol/L microcystin-LR, 10 mmol/L sodium orthovanadate, and 2 μg/mL each of pepstatin, antipain, and leupeptin] for immunoprecipitation. IRβ, p85, IRS-1, and IRS-2 were immunoprecipitated from lysates as previously described.^24,26^ Immunoprecipitates and whole cell lysates were analysed by SDS–PAGE/western blotting using 6% Tris-glycine and 8% bis-Tris gels as previously described.^24,27^ For quantification of immunoblotting, densitometry was performed using the ImageJ software (NIH).

### 2.8 Quantification of IGF release

IGF-1 and IGF-2 concentrations in platelet releasates were measured using an IGF-1 mouse/rat quantikine ELISA kit (R&D Systems™) or an IGF-2 mouse ELISA kit (Abcam^®^) following the manufacturer's protocol. Releasates were prepared by incubating 100 µL of platelets (4 × 10^8^/mL) with CRP-XL or PAR4-AP as indicated. Platelets were then removed by centrifugation (520 ×*g*, 5 min × 2).

### 2.9 Statistical analyses

All data are presented as the mean ± S.E.M. of at least three independent observations. Data presented with statistical analysis were tested using a Student's *t*-test or two-way ANOVA with Bonferroni post-tests for multiple comparison analysis. Significance was set as *P* < 0.05.

## 3. Results

### 3.1 Insulin signalling in mouse platelets

**Figure 1:**
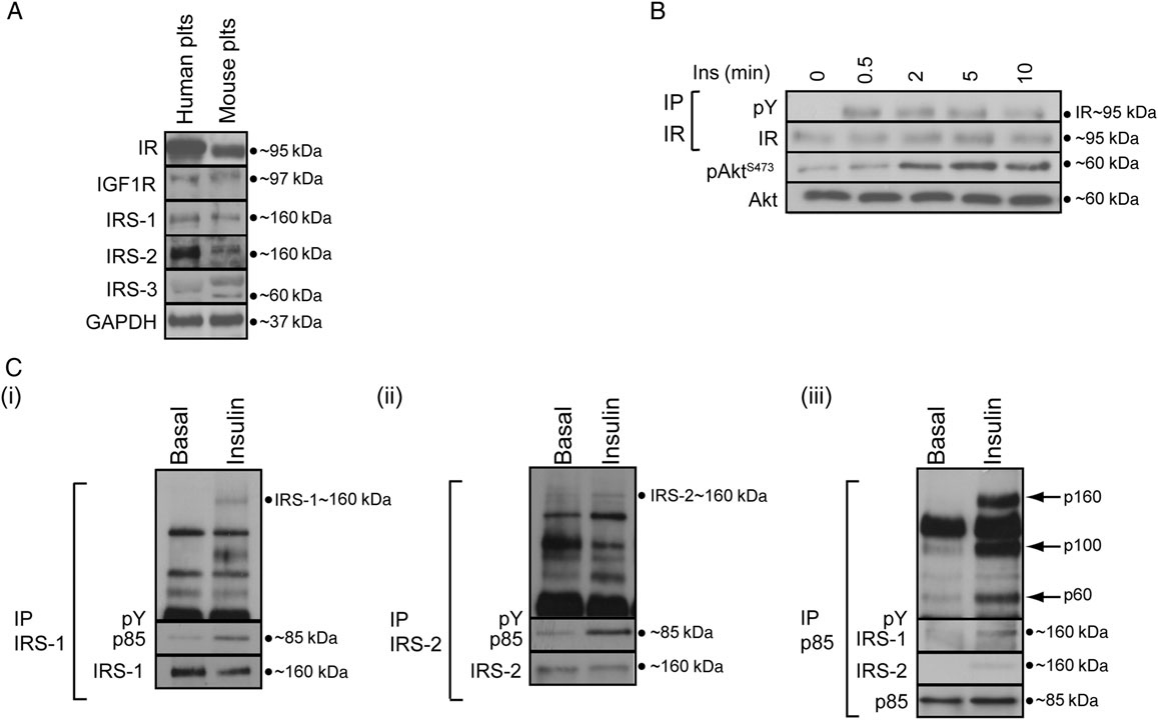
Mouse platelets express functional IRs. (*A*) Immunoblotting demonstrated that IR, IGF1R, IRS-1, and IRS-2 are expressed in human and mouse platelets. IRS-3 was expressed only in mouse platelets (*n* = 5). (*B*) Time course of insulin (20 nM) stimulation of mouse platelets showing tyrosine phosphorylation of IR and the PI3K effector Akt (Ser^473^; *n* = 3). (*C*) Insulin (20 nM, 5 min) stimulation of mouse platelets leads to tyrosine phosphorylation of IRS-1 (i) and IRS-2 (ii) and induces their association with the PI3K regulatory subunit p85 (i–iii) (*n* = 3). Furthermore, tyrosine-phosphorylated (pY) proteins at 160, 100, and 60 kDa (see arrows) were found to be associated with p85 after insulin stimulation (iii). Phosphorylation of the indicated proteins was assessed by western blotting of either whole cell lysates or immunoprecipitates (IP). Membranes were reprobed to confirm equal loading.

We, and others, previously demonstrated that human platelets express IR, IGF1R, and the substrates IRS-1 and 2.^24,26,28^ In agreement with these data, we find that mouse platelets (IR^flox/flox^ *Pf4*-Cre^−^) also express IR, IGF1R, and the substrates IRS-1, 2, and 3 (*Figure 1A*). Insulin stimulated rapid tyrosine phosphorylation of IR, phosphorylation of the PI3K effector Akt at Ser473 (*Figure 1B*), and tyrosine phosphorylation of IRS-1 and IRS-2, and their association with the regulatory PI3K subunit p85 (*Figure 1C*). Insulin also induced the association of tyrosine-phosphorylated proteins (160, 100, and 60 kDa) with p85.

### 3.2 IR-deficient platelets no longer respond to insulin

**Figure 2:**
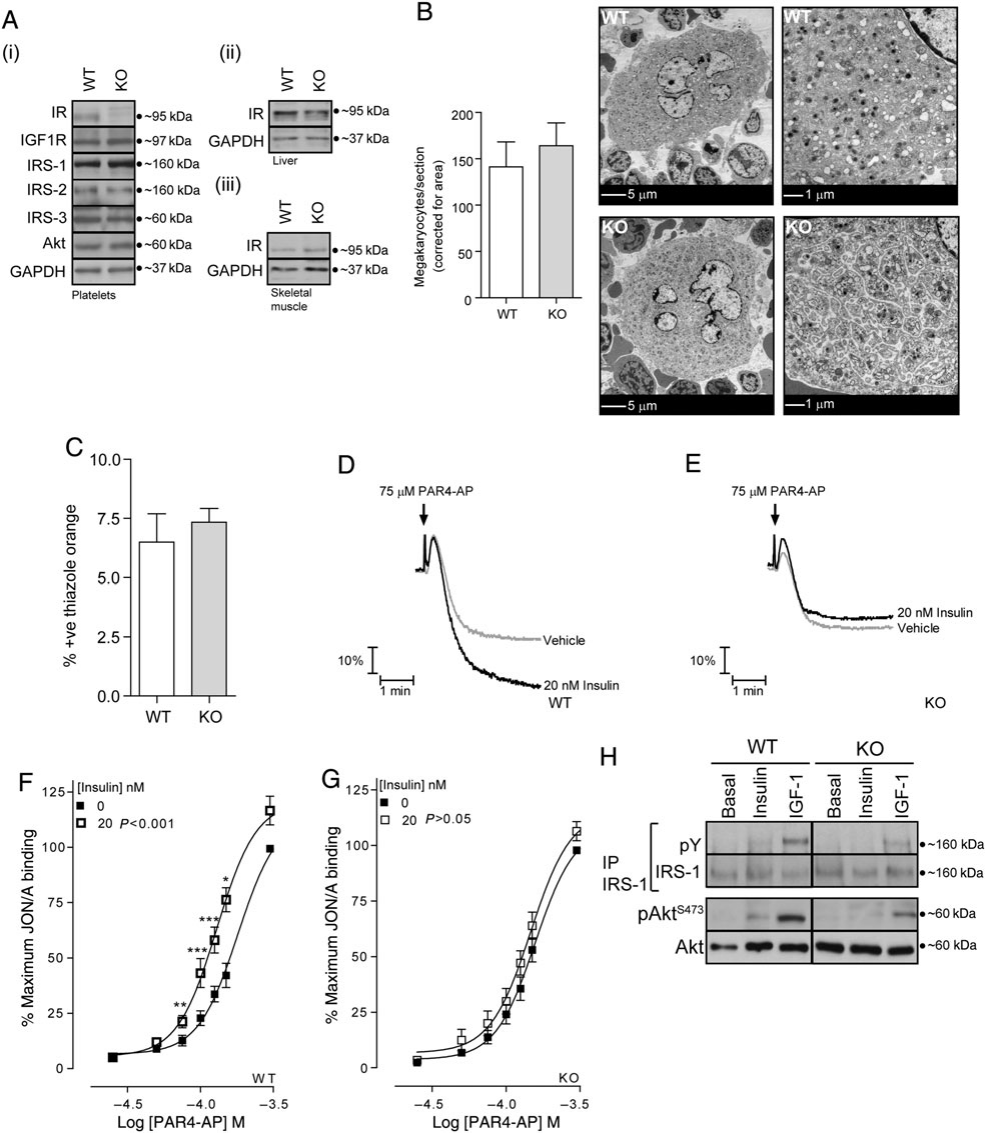
Insulin acting at IR enhances mouse platelet function. (*A*) Immunoblotting confirmed ablation of IR protein expression in platelets (i) but not in liver (ii) or skeletal muscle (iii) from IR^flox/flox^; *Pf4*-Cre^+^ (KO) mice. KO platelet expression of IGF1R, IRS-1, 2, 3, and Akt protein were comparable to WT platelets (i, *n* = 3). (*B*) For evaluation of megakaryocyte number and morphology sections of bone marrow were imaged with a transmission electron microscope. The number of megakaryocytes per section was quantified manually using ImageJ, and expressed as the number of megakaryocytes/section corrected for total area of the section. Bars represent means ± SE (*n* = 3). Data were analysed by Student's unpaired *t*-test, and there was no significant difference in the number of megakaryocytes in WT and KO mice. Subjectively, there was no difference in the subcellular morphology of WT megakaryocytes compared with those lacking IR. Scale bars = 5 and 1 μm. (*C*) For evaluation of reticulated platelets by flow cytometry, whole blood from WT and KO mice was fixed, stained for CD61-PE, and incubated with thiazole orange (Retic-count™, BD Bioscience). Reticulated platelets are expressed as a percentage of thiazole orange and CD61-PE double-positive cells in 10 000 identified platelets. Bars represent mean ± SE (*n* = 3). Data were analysed by Student's unpaired *t*-test, and there was no significant difference in the number of reticulated platelets in WT and KO mice. (*D* and *E*) Monitoring of platelet aggregation using a Chronolog 590-2A aggregometer demonstrated that pre-incubation of platelets (2 × 10^8^/mL) with insulin (20 nM, 5 min) enhances PAR-4-mediated platelet aggregation in WT (*D*), but not in IR KO (*E*) platelets (*n* = 3). (*F* and *G*) In agreement with platelet aggregation, PAR-4-mediated integrin α_IIb_β_3_ activation, as measured by FACS using the JON/A antibody against the high affinity integrin α_IIb_β_3_ conformation, was enhanced by insulin (20 nM, 5 min) in WT (*F*) but not in IR KO platelets (*G*). FACS results are expressed as mean ± SE of the percentage of maximal fluorescence of vehicle-treated platelets (*n* = 13). Data were analysed by two-way ANOVA (*P*-value), followed by Bonferroni's post-test (asterisks). A value of *P* < 0.05 indicates that insulin significantly altered PAR-4-mediated integrin activation in WT platelets. Asterisks (*P < 0.05, **P < 0.01, and ***P < 0.001) indicate a significant difference induced by insulin from vehicle at the matching PAR4-AP concentration. (*H*) Immunoprecipitation of IRS-1 demonstrates that IGF-1 (100 nM, 5 min), but not insulin (20 nM, 5 min), stimulates tyrosine phosphorylation (pY) of IRS-1 in IR KO platelets (*n* = 3). Membranes were reprobed to confirm equal loading.

*Figure 2A* demonstrates that we successfully generated a mice line deficient in platelet IR (IR^flox/flox^ *Pf4*-Cre^+^), whereas the levels of key signalling components such as IGF1R, IRS-1, IRS-2, IRS-3, and Akt were comparable to WT platelets. Furthermore, IR^flox/flox^ *Pf4*-Cre^+^ mice expressed normal levels of IR in liver and skeletal muscle (*Figure 2A*ii and iii). Deletion of IR resulted in increased platelet counts and mean platelet volumes from 785 ± 17 to 845 ± 20 × 0^3^/µL (*P* < 0.05, *n* = 28) and 5.06 ± 0.03 to 5.18 ± 0.04 µm^3^ (*P* < 0.05, *n* = 28), respectively. This was not accompanied by alterations in the number or morphology of the megakaryocytes (*Figure 2B*) or the percentage of thiazole orange-positive (reticulated) platelets (*Figure 2C*). White and red blood cell counts were not significantly altered.

Insulin (20 nM) significantly increased platelet aggregation induced by the predominant murine thrombin receptor PAR-4 in WT mouse platelets (*Figure 2D*). This correlated with an increase in the activation of the integrin α_IIb_β_3_ by PAR4-AP (*Figure 2F*). Insulin-mediated enhancement of integrin activation was observed with insulin concentrations ranging from 0.5 to 100 nM (data not shown). Loss of the IR resulted in an ablation of the enhancing effect of insulin (*Figure 2E* and *G*), demonstrating a loss of insulin sensitivity. Furthermore, IGF-1, but not insulin, induced tyrosine phosphorylation of IRS-1 and pSer^473^ Akt (*Figure 2H*) in platelets lacking the IR.

### 3.3 Impaired platelet functional responses downstream of GPVI in the absence of IR

**Figure 3:**
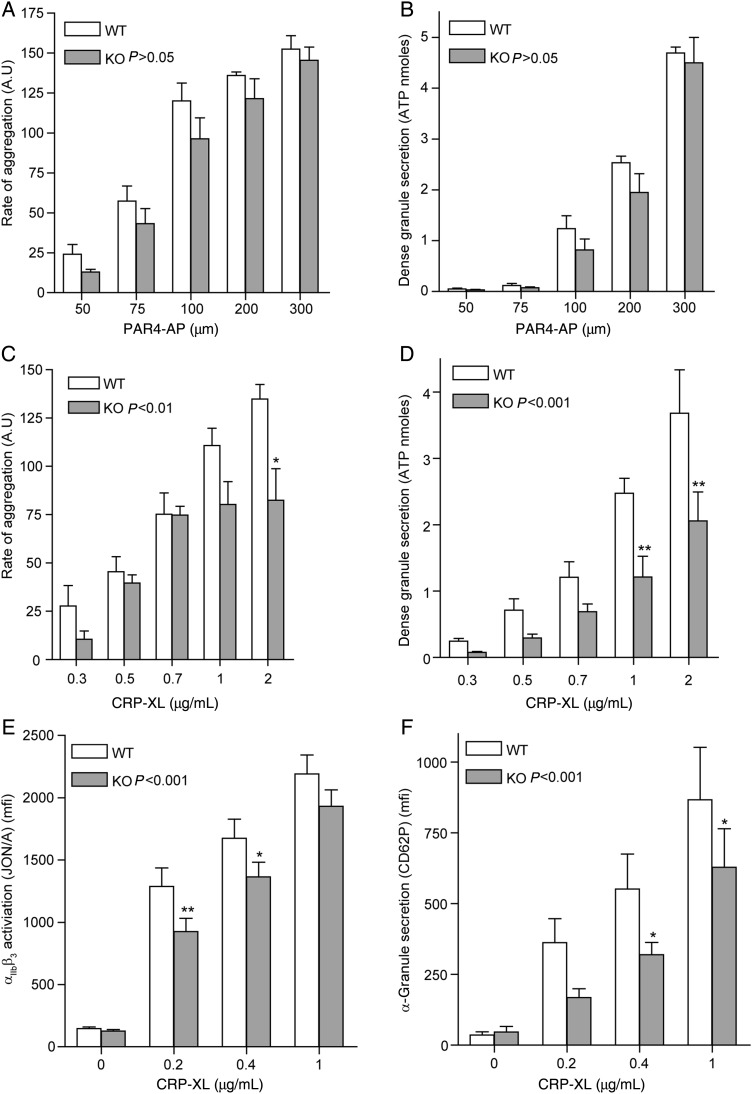
Deletion of IR results in a reduction in GPVI-mediated platelet activation. (*A* and *B*) Monitoring of platelet aggregation and dense granule secretion using a luciferin/luciferase reagent (Chronolume) in a Chronolog 590-2A aggregometer demonstrated that PAR4-AP-mediated platelet aggregation (*A*, *n* = 11) and dense granule secretion (*B*, *n* = 11) were not significantly altered in IR KO platelets. (*C* and *D*) In contrast, CRP-XL-mediated platelet aggregation (*C*, *n* = 14) and dense granule secretion (*D*, *n* = 8) were significantly reduced in IR KO platelets. (*E* and *F*) Furthermore, CRP-XL-mediated integrin α_IIb_β_3_ activation (*E*, *n* = 9) and α-granule secretion (*F*, *n* = 9) measured by FACS using JON/A and CD62P antibodies, respectively, were significantly reduced in IR KO platelets. Results are expressed as mean ± SE of either the rate of aggregation, ATP nmoles, or mean fluorescence intensity. Data were analysed by two-way ANOVA (*P*-value), followed by Bonferroni's post-test (asterisks). A value of *P* < 0.05 indicates that CRP-XL-mediated aggregation, dense and α-granule secretion, and integrin activation were significantly different in IR KO platelets in comparison with WT. Asterisks (*P < 0.05, **P < 0.01) indicate a significant difference from WT at the matching CRP-XL concentrations.

To investigate whether insulin resistance affects platelet activity, we analysed platelet activation by PAR-4 and the collagen receptor GPVI in IR-deficient platelets. PAR-4-mediated platelet aggregation (*Figure 3A*) and dense granule secretion (*Figure 3B*) were not significantly affected in IR-deficient platelets. In contrast, CRP-XL-mediated aggregation and dense granule secretion were significantly reduced with an approximate 50% reduction in ATP released in response to CRP-XL at a range of concentrations (*Figure 3C* and *D*). Furthermore, we observed analogous reductions in both CRP-XL-mediated integrin α_IIb_β_3_ activation and α-granule secretion (*Figure 3E* and *F*). This impairment was not due to a reduction in GPVI levels, as platelet surface expression of GPVI analysed by FACS was comparable between WT and KO platelets (1287 ± 40 and 1214 ± 37 mfi, respectively, *n* = 21).

### 3.4 Reduction in GPVI-mediated signalling in IR-deficient platelets

**Figure 4:**
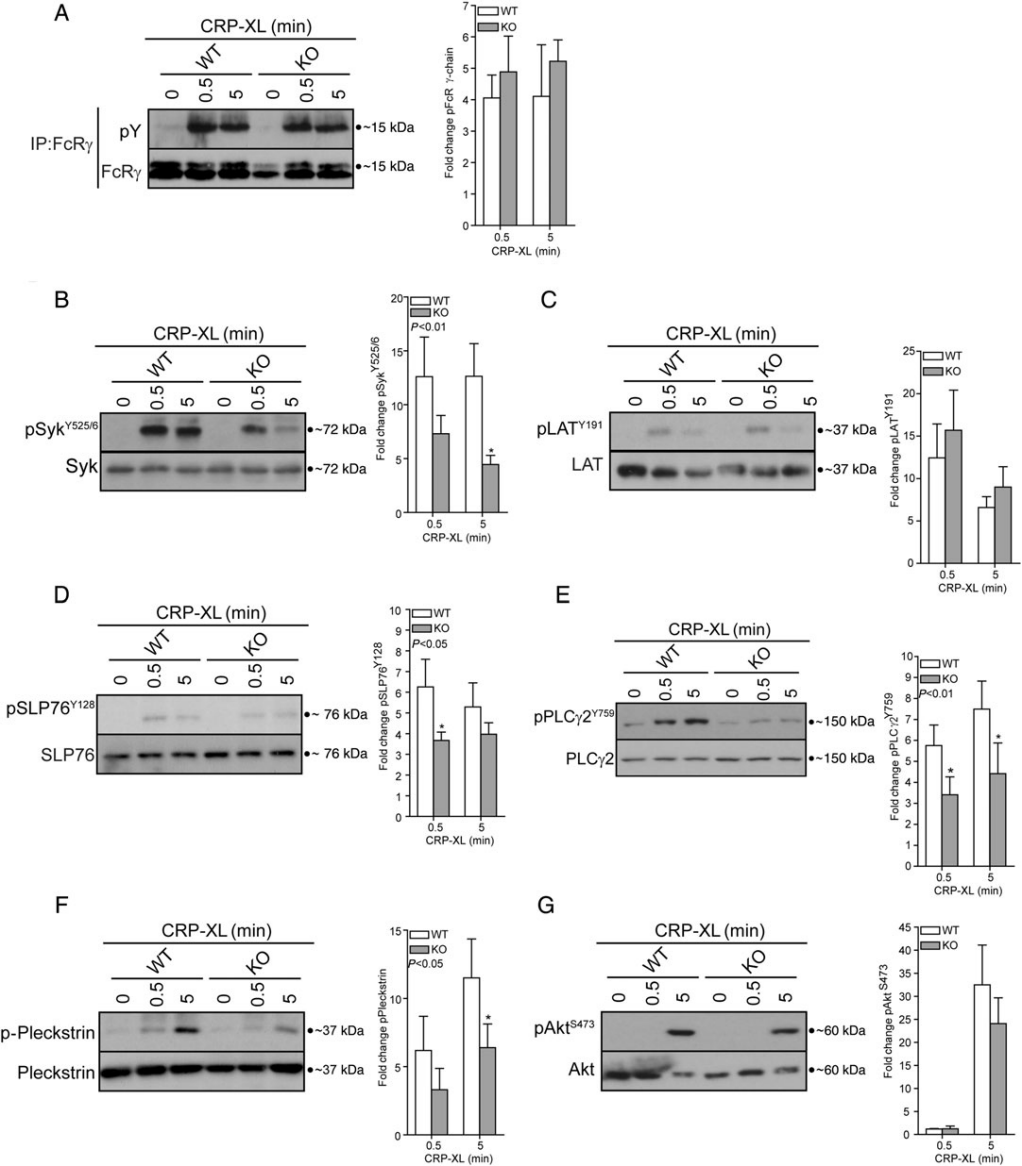
Deletion of IR results in a reduction in GPVI signalling. Stimulation of platelets with CRP-XL (1 µg/mL) induced phosphorylation of (*A*) FcR γ-chain (IP: FcR γ-chain, IB:4G10, *n* = 3), (*B*) Syk (Tyr^525/6^, *n* = 7), (*C*) LAT (Tyr^191^, *n* = 9), (*D*) SLP-76 (Tyr^128^, *n* = 8), (*E*) PLCγ2 (Tyr^759^, *n* = 6), (*F*) pleckstrin (PKC substrates antibody, *n* = 7), and (*G*) Akt (Ser^473^, *n* = 9). The deletion of IR resulted in a significant reduction in Syk, SLP-76, PLCγ2, and pleckstrin phosphorylation (*B* and *D*–*F*). Bars represent means ± SE of phosphorylation (ratio phosphorylated/total), expressed as a fold change from unstimulated conditions (CRP-XL, *t* = 0). Data were analysed by two-way ANOVA (*P*-value), followed by Bonferroni's post-test (asterisks). A value of *P* < 0.05 indicates that CRP-XL-mediated Syk, SLP-76, PLCγ2, and pleckstrin phosphorylation were significantly different in IR KO platelets in comparison with WT. Asterisks (*P < 0.05) indicate a significant difference from WT at the matching time point. Phosphorylation of the indicated proteins was assessed by western blotting of either whole cell lysates or immunoprecipitates (IP). Membranes were reprobed to confirm equal loading.

We next investigated whether GPVI-mediated signalling events were affected in IR-deficient platelets. Stimulation of platelets with CRP-XL (1 µg/ml) for either 0.5 or 5 min resulted in tyrosine phosphorylation of FcR γ-chain, Syk (Tyr^525/6^), LAT (Tyr^191^), SLP-76 (Tyr^128^), PLCγ2 (Tyr^759^), the PKC substrate pleckstrin, and Akt (Ser^473^) (*Figure 4*A–*F*). In comparison with WT platelets, there was a general decrease in GPVI-mediated signalling events downstream of GPVI and FcR γ-chain phosphorylation with significant reductions in Syk (Tyr^525/6^), SLP-76 (Tyr^128^), PLCγ2 (Tyr^759^), and pleckstrin phosphorylation (*Figure 4A*, *D*, and *E*). Surprisingly, although LAT is a substrate of Syk, its phosphorylation was unaffected. PAR-4 (100 µM, 0.5 and 5 min)-mediated phosphorylation of Ser^473^ Akt and pleckstrin was not significantly altered in KO platelets (*n* = 8, data not shown).

### 3.5 Loss of IR results in a reduction in IGF-1 and IGF-2 signalling

**Figure 5:**
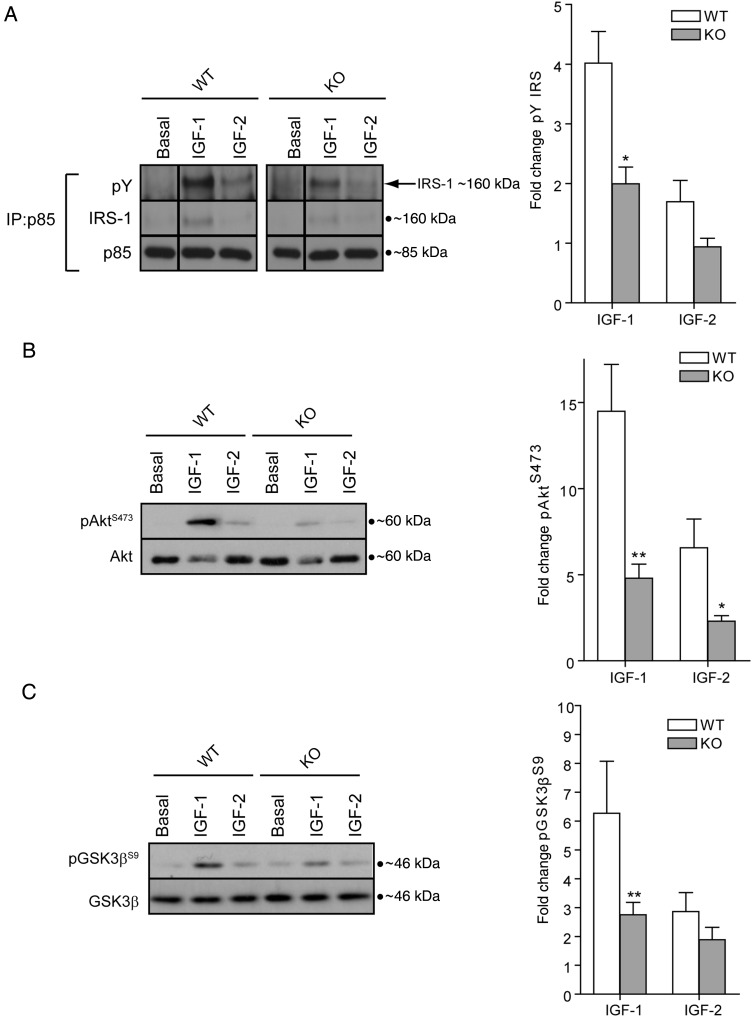
Reduction in IGF-1 and IGF-2 signalling in IR-deficient platelets. Stimulation of platelets (4 × 10^8^/mL) revealed a significant reduction in IGF-1 (100 nM, 5 min) and IGF-2 (100 nM, 5 min) induced phosphorylation of (*A*) IRS-1 (IP:p85, IB:4G10, and IRS-1, *n* = 3), (*B*) Akt [Ser^473^, *n* = 13 (IGF-1)/8 (IGF-2)], and (*C*) GSK3β (Ser^9^, *n* = 6). Bars represent means ± SE of phosphorylation (ratio phosphorylated/total), expressed as a fold change from basal. Data were analysed by Student's unpaired *t*-test. Asterisks (*P < 0.05, **P < 0.01) indicate a significant difference from matched WT stimulation.

Genetic deletion of IR will result not only in the loss of IR homodimers but also of IR/IGF1R heterodimers. These IR/IGF1R heterodimers are thought to play a predominant role in mediating IGF-1s signalling in platelets and support of platelet function.^26,29^ IR/IGFR heterodimers have a high affinity for IGF-1, a lower affinity for IGF-2, and low/insignificant affinity for insulin.^30^ IGF-2 can furthermore activate IR-A homodimer receptors.^31^ IGF-1 and IGF-2 stimulate association of tyrosine-phosphorylated IRS-1 with p85 (*Figure 5A*) and phosphorylation of Akt (Ser^473^, *Figure 5B*) and GSK3β (Ser^9^, *Figure 5C*). IGF-2 induced weaker phosphorylation than IGF-1, similar to the results seen with insulin (*Figure 2F*). Loss of IR resulted in a marked reduction in phosphorylation of IRS-1, Akt (Ser^473^), and GSK3β (Ser^9^) induced by both growth factors (*Figure 5A*–*C*). These data suggest that IR homodimers and/or IR/IGF1R hybrids are required for maximal activation of signalling pathways in platelets by IGF-1 and IGF-2.

### 3.6 Impaired GPVI-mediated platelet function in the presence of IGF-1 and IGF-2

**Figure 6:**
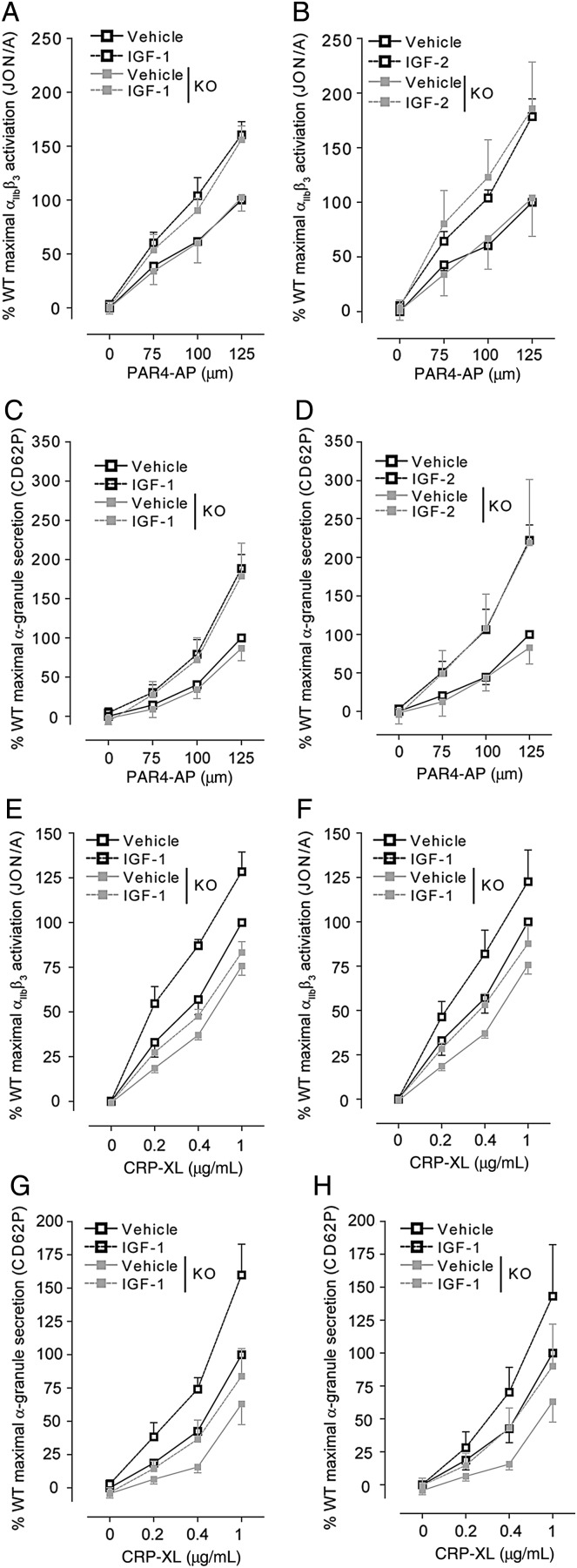
Reduction in CRP-XL, but not in PAR-4 platelet activation, in the presence of IGF-1 and IGF-2. IGF-1 (*n* = 6) and IGF-2 (*n* = 4) enhanced PAR-4-mediated integrin α_IIb_β_3_ activation (*A* and *B*) and α-granule secretion (*C* and *D*) to a similar degree in IR KO compared with WT. In contrast, CRP-XL in the presence of IGF-1 (*n* = 3) or IGF-2 (*n* = 3) failed to induce similar levels of integrin α_IIb_β_3_ activation (*E* and *F*) or α-granule secretion (*G* and *H*) in KO platelets compared with WT. Integrin α_IIb_β_3_ activation and α-granule secretion were measured by FACS using the JON/A and CD62P antibodies, respectively. Results are expressed as mean ± SE of the percentage of maximal fluorescence of vehicle-treated WT platelets. Data were analysed by two-way ANOVA, followed by Bonferroni's post-test.

Owing to the decrease in IGF-1/2 signalling, we examined whether the effect of IGF-1/2 on platelet function was also compromised in IR-deficient platelets. Stimulation of platelets with PAR4-AP (*Figure 6A*–*D*) or CRP-XL (*Figure 6E*–*H*) in the presence of maximal concentrations of IGF-1 or IGF-2 resulted in a significant increase in both PAR-4 and GPVI-mediated integrin α_IIb_β_3_ activation (*Figure 6A*, *B*, *E*, and *F*) and α-granule secretion (*Figure 6C*, *D*, *G*, and *H*). Increases induced by either IGF-1 or IGF-2 were comparable. Loss of IR did not result in significant alterations in platelet activation induced by PAR-4 in the presence of either IGF-1 or IGF-2 (*Figure 6A*–*D*). In contrast, integrin α_IIb_β_3_ activation and α-granule secretion induced by CRP-XL in presence of IGF-1 (*P* < 0.001, *n* = 3) or IGF-2 (*P* < 0.001, *n* = 3) were significantly reduced in IR-deficient platelets (*Figure 6E*–*H*).

### 3.7 Pharmacological inhibition of IR/IGF1R inhibits GPVI-mediated platelet function

**Figure 7:**
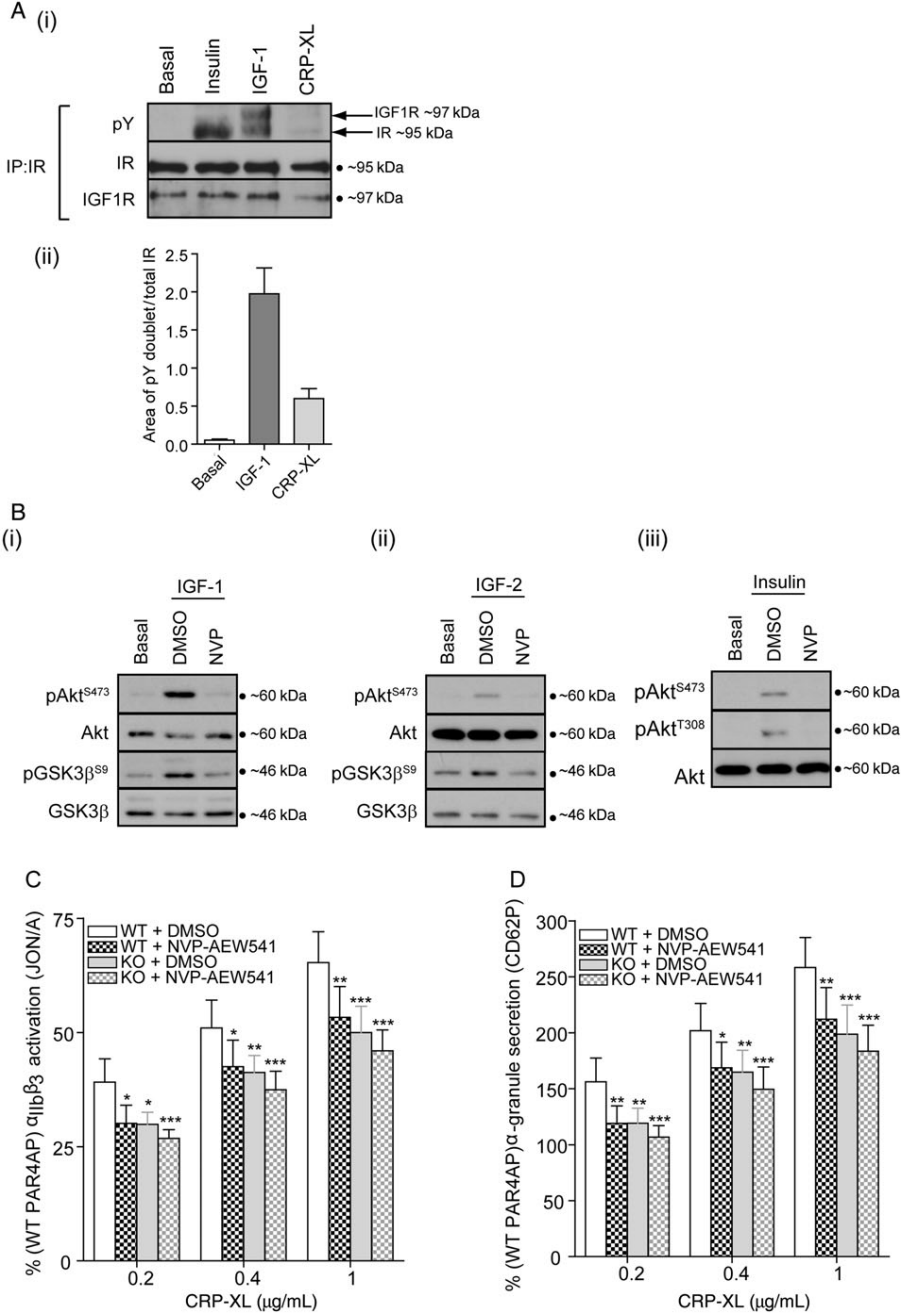
Pharmacological inhibition of IR/IGF1R reduces GPVI platelet activation to levels comparable to IR-deficient platelets. (*A*, i) Immunoprecipitation of IR from platelet lysates followed by immunoblotting with 4G10 (pY), IR, and IGF1R antibodies demonstrated that IGF-1 (100 nM, 5 min) and CRP-XL (5 µg/ml, 5 min) can induce tyrosine phosphorylation of IR/IGF1R heterodimers (*n* = 4). Bars (ii) represent means ± SE of doublet phosphorylation (ratio phosphorylated/IR total). (*B*) Immunoblotting demonstrated that pre-incubation of platelets with NVP-AEW541 (1 µM, 10 min) blocked IGF-1 (i, 100 nM, 5 min), IGF-2 (ii, 100 nM, 5 min), and insulin (iii, 20 nM, 5 min) induced phosphorylation of Akt (Ser^473^, Thr^308^) and GSK3β (Ser^9^) (*n* = 6, 4, and 3, respectively). Pre-incubation of platelets with NVP-AEW541 (1 µM, 10 min) resulted in a significant reduction in WT, but not in KO CRP-XL-mediated integrin α_IIb_β_3_ activation (*C*) and α-granule secretion (*D*) measured by FACS using the JON/A and CD62P antibodies, respectively. Results are expressed as mean ± SE of the percentage of PAR-4 response (125 µM) (*n* = 5). Data were analysed by two-way ANOVA, followed by Bonferroni's post-test. Asterisks (*P < 0.05, **P < 0.01, ***P < 0.001) indicate a significant difference from WT + DMSO at the matching concentration.

Our data suggest that maximal GPVI-mediated platelet activation requires the tyrosine kinase activity of IR and/or IR/IGF1R. Immunoprecipitation of IR demonstrated that IR/IGF1R hybrids are present in mouse platelets, and that these are tyrosine-phosphorylated by CRP-XL (*Figure 7A*i). Although CRP-XL-induced phosphorylation was considerably weaker compared with addition of a maximal concentration of exogenous IGF-1, these data reflect that stimulation of platelets with CRP-XL can activate IR/IGF1R heterodimers (*Figure 7A*i and ii).

To confirm that IR/IGF1R activity is required to support GPVI-mediated platelet activation, we used NVP-AEW541, a potent inhibitor of IR and IGF1R kinase activity. Signalling downstream of IGF-1, IGF-2, and insulin was ablated by NVP-AEW541 (*Figure 7B*), confirming that this inhibitor can block IR and IGF1R activity in mouse platelets. Treatment of platelets with NVP-AEW541 resulted in a significant inhibition of GPVI-mediated integrin α_IIb_β_3_ activation and α-granule secretion (*Figure 7C* and *D*) in WT but not in KO platelets. Furthermore, GPVI-mediated platelet activation in NVP-AEW541-treated WT platelets was not significantly different from IR-deficient platelets in the absence or presence of NVP-AEW541 (*Figure 7C* and *D*). In contrast, PAR-4 (125 µM)-mediated integrin α_IIb_β_3_ activation and α-granule secretion in WT platelets were not significantly reduced by NVP-AEW541 (mfi = 1127 ± 223–1192 ± 237 and 292 ± 66–315 ± 67, respectively, *n* = 5). Therefore, pharmacological inhibition of IR/IGF1R activity can recapitulate the platelet activation phenotype of IR-deficient platelets in WT platelets. Taken together, these results conclusively demonstrate that IR/IGF1R kinase activity is required to support GPVI-mediated platelet function, and that either deletion of IR or pharmacological inhibition of IR/IGF1R kinase activity results in impaired GPVI-mediated platelet function.

## 4. Discussion

We originally hypothesized that megakaryocyte/platelet insulin resistance would result in platelet hyperactivity. Conversely, we found that platelet activation was unchanged in response to PAR-4 and reduced in response to GPVI. Several previous studies suggested that insulin resistance of megakaryocytes/platelets is the underlying cause of the platelet hyperactivity observed in patients with insulin resistance.^17,18^ Here, we demonstrate that deletion of IR, using IR^flox/flox^; Pf4-Cre mice, did not increase platelet activation in response to either the PAR-4 agonist PAR4-AP or GPVI agonist CRP-XL. Furthermore, whole blood thrombus formation on collagen was also unaffected (*n* = 5, data not shown). The lack of effect is unlikely to be due to species differences as rodents have been reported to acquire a similar platelet hyperactive phenotype associated with insulin resistance.^32–34^ Our results therefore demonstrate that the increase in platelet activity observed in conditions/diseases associated with insulin resistance is unlikely to be due to a loss of megakaryocyte/platelet IR signalling.

Interestingly, IR KO mice had elevated platelet counts and increased mean platelet volumes, which are frequently reported in patients with obesity and T2DM,^35,36^ suggesting that megakaryocyte/platelet insulin resistance can contribute to the changes in platelet count and volume in obesity and T2DM. Although it has been suggested that the increase in mean platelet volume in patients with T2DM/metabolic syndrome^37,38^ is due to an increased turnover of the platelet population, we found no significant alterations in the percentage of circulating reticulated platelets. Furthermore, we observed no significant alterations in megakaryocyte number or morphology, despite a previous study demonstrating that insulin altered murine megakaryopoiesis *in vitro*, enhancing the number of immature megakaryocytes present in cultures.^33^

Deletion of IR resulted in a significant and specific decrease in GPVI function in mouse platelets. Furthermore, we found that CRP-XL induced weak tyrosine phosphorylation of IR/IGF1R heterodimers, suggesting that these hybrid receptors may play a role in supporting GPVI-mediated platelet function. One of the mechanisms by which the tyrosine kinase activity of the IR/IGF1R hybrids could be activated is by the release of IGFs from activated platelets.^39–41^ IGF-1 has been previously demonstrated to amplify platelet activation by autocrine/paracrine effects through IR/IGFR homodimer and heterodimer receptors.^26,29^ We also report here for the first time that IGF-2 is able to enhance platelet function. Deletion of IR will result in a loss of both IR/IR and IR/IGF1R receptors and may thereby reduce the effect of IGF-1 and IGF-2 on platelet function. We indeed found a reduction in IGF1- and IGF2-mediated phosphorylation of IRS-1, Akt, and GSK3 in IR KO platelets, suggesting that under normal conditions IGF-1/-2 signalling is predominantly through IR homodimers and IR/IGF1R heterodimers. This was associated with a reduced priming effect of IGF-1 and IGF-2 on GPVI-mediated platelet function. The reduction in GPVI-mediated platelet function in IR KO may therefore be due to impairment in the effect of released IGFs on platelet function. In support of this, we found that inhibition of IR and IGFR activity using NVP-AEW541 reduced GPVI-mediated platelet function in WT platelets to the same extent as IR deletion, but had no effect on IR KO platelets.

Interestingly, however, PAR4-AP-mediated platelet function was not affected by IR deletion or the IR/IGFR kinase inhibitor NVP-AEW541, and we were unable to detect IR/IGFR hybrid phosphorylation by PAR4-AP, demonstrating that IR/IGFR activity and/or released IGF does not contribute to PAR4-mediated platelet function under these conditions.

One possible explanation is that CRP-XL is more potent at releasing IGF1/2 than PAR4-AP. We indeed found that CRP-XL (5 µg/mL, 5 min) induced more release of IGF-1 and IGF-2 (44.3 ± 7.25 and 143.3 ± 62.2 pM, respectively, increase from basal per 5 × 10^7^ platelets, *n* = 4) than PAR4-AP (26.0 ± 0.9 and 83.0 ± 48.85 pM, respectively, increase from basal per 5 × 10^7^ platelets, *n* = 3). Although the concentrations are lower than used *in vitro*, endogenous IGF1/2 from the α-granules is released near the membrane and is therefore likely to be more efficient. Potentially PAR-4 function/signalling pathways are less reliant on IGF signalling than GPVI pathways, with the threshold for maximal PAR4-mediated activation still being achieved in the IR KO mice. Note that we were unable to use receptor and/or IGF1/2 blocking antibodies to confirm a role for released IGF1/2, as control studies showed that they did not block the effect of IGF1/2 on murine platelets. We can therefore not completely exclude the possibility that CRP activates IR/IGFR hybrid receptors through an alternative mechanism, for example through ligand-independent trans-phosphorylation/activation of IR/IGF1R heterodimers.^42^

The observation that insulin significantly enhanced mouse platelet activation is interesting, as it demonstrates that signalling downstream of IR homodimers increases platelet function. We previously reported that, in human platelets, insulin had minimal effects on platelet functional responses,^24^ as the majority of IR subunits are expressed as IR/IGF1R heterodimers, which are relatively unresponsive to insulin.^26,43^ In contrast, insulin significantly enhanced mouse platelet activation and stimulated strong phosphorylation of IR homodimers, phosphorylation of IRS-1 and IRS-2, and activation of PI3K, demonstrating that activation of IR homodimers increases platelet function.

Taken together, our data demonstrate that megakaryocyte/platelet insulin resistance may contribute to increases in platelet number and volume, but not in platelet hyperactivity, in insulin-resistant patients. Furthermore, insulin, IGF-1 and IGF-2 are able to enhance mouse platelet activation and a loss in IR/IGF1R activity impacts on GPVI-mediated signalling and function in platelets.

## Funding

This work was supported by the British Heart Foundation (grant nos PG/08/056/25325, PG/10/100/28658, FS/12/3/29232, and PG/14/3/30565). Funding to pay the Open Access publication charges for this article was provided by the Charities Open Access Fund (COAF), University of Bristol.
